# *catena*-Poly[[di­aqua­bis­(4-formyl­benzo­ato-κ*O* ^1^)nickel(II)]-μ-pyrazine-κ^2^ *N*:*N*′]

**DOI:** 10.1107/S160053681400155X

**Published:** 2014-01-25

**Authors:** Fatih Çelik, Nefise Dilek, Nagihan Çaylak Delibaş, Hacali Necefoğlu, Tuncer Hökelek

**Affiliations:** a Department of Chemistry, Kafkas University, 36100 Kars, Turkey; b Aksaray University, Department of Physics, 68100, Aksaray, Turkey; c Department of Physics, Sakarya University, 54187 Esentepe, Sakarya, Turkey; d Department of Physics, Hacettepe University, 06800 Beytepe, Ankara, Turkey

## Abstract

In the title polymeric compound, [Ni(C_8_H_5_O_3_)_2_(C_4_H_4_N_2_)(H_2_O)_2_]_*n*_, the Ni^II^ atom is located on a twofold rotation axis and has a slightly distorted octa­hedral coordination sphere. In the equatorial plane, it is coordinated by two carboxyl­ate O atoms of two symmetry-related monodentate formyl­benzoate anions and by two N atoms of the bridging pyrazine ligand, which is bis­ected by the twofold rotation axis. The axial positions are occupied by two O atoms of the coordinating water mol­ecules. In the formyl­benzoate anion, the carboxyl­ate group is twisted away from the attached benzene ring by 7.0 (6)°, while the benzene and pyrazine rings are oriented at a dihedral angle of 66.2 (3)°. The pyrazine ligands bridge the Ni^II^ cations, forming polymeric chains running along the *b*-axis direction. Intra­molecular O—H⋯O hydrogen bonds link the water ligands to the carboxyl­ate O atoms. In the crystal, water–water O—H⋯O hydrogen bonds link adjacent chains into layers parallel to the *bc* plane. Pyrazine–formyl C—H⋯O hydrogen bonds link the layers, forming a three-dimensional network. There are also weak C—H⋯π inter­actions present. The title compound is isotypic with the copper(II) complex [Çelik *et al.* (2014*a*). *Acta Cryst*. E**70**, m4–m5].

## Related literature   

For the structural functions and coordination relationships of the aryl­carboxyl­ate ion in transition-metal complexes of benzoic acid derivatives, see: Nadzhafov *et al.* (1981 ▶); Shnulin *et al.* (1981 ▶). For applications of transition-metal complexes with biochemical mol­ecules in biological systems, see: Antolini *et al.* (1982 ▶). Some benzoic acid derivatives, such as 4-amino­benzoic acid, have been extensively reported in coordination chemistry, as bifunctional organic ligands, due to the varieties of their coordination modes, see: Chen & Chen (2002 ▶); Amiraslanov *et al.* (1979 ▶); Hauptmann *et al.* (2000 ▶). For the isotypic copper(II) complex, see: Çelik *et al.* (2014*a* ▶). For other related structures involving 4-formyl­benzoate, see: Çelik *et al.* (2014*b* ▶); Hökelek *et al.* (2009 ▶). For standard bond lengths, see: Allen *et al.* (1987 ▶).
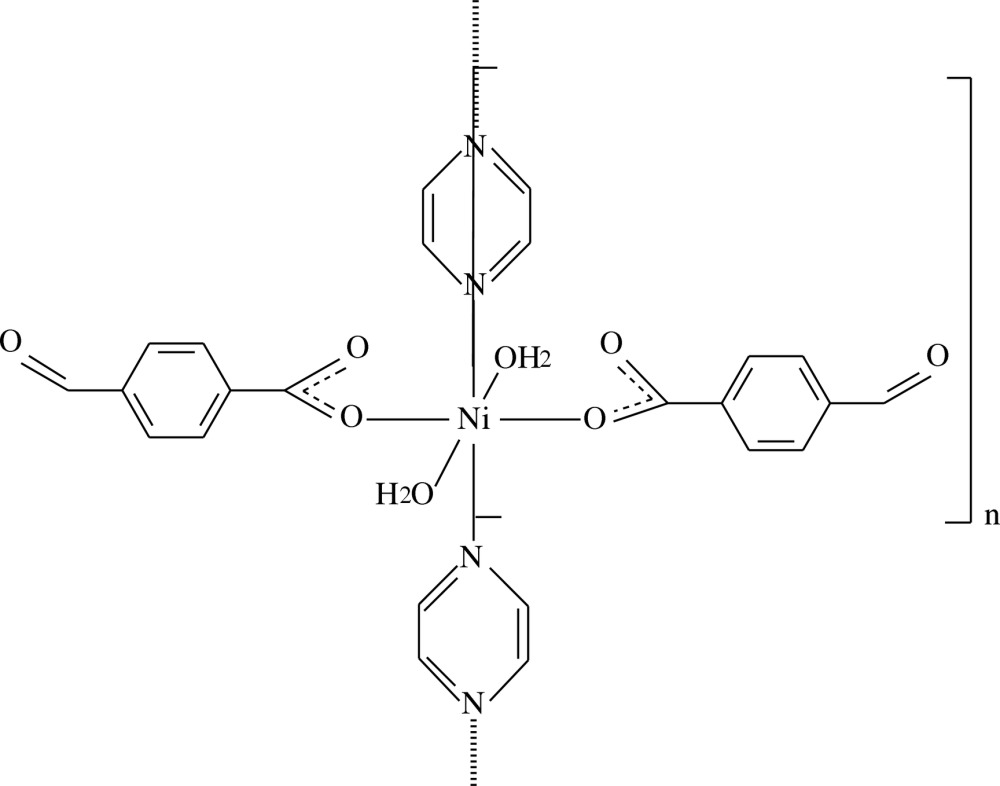


## Experimental   

### 

#### Crystal data  

[Ni(C_8_H_5_O_3_)_2_(C_4_H_4_N_2_)(H_2_O)_2_]
*M* *_r_* = 473.07
Monoclinic, 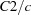

*a* = 22.1032 (5) Å
*b* = 6.9925 (2) Å
*c* = 12.3366 (3) Å
β = 94.160 (3)°
*V* = 1901.68 (8) Å^3^
*Z* = 4
Mo *K*α radiation
μ = 1.08 mm^−1^
*T* = 296 K
0.48 × 0.23 × 0.14 mm

#### Data collection  

Bruker SMART BREEZE CCD diffractometer
Absorption correction: multi-scan (*SADABS*; Bruker, 2012 ▶) *T*_min_ = 0.743, *T*_max_ = 0.860
9913 measured reflections
1717 independent reflections
1554 reflections with *I* > 2σ(*I*)
*R*_int_ = 0.070

#### Refinement  

*R*[*F* ^2^ > 2σ(*F* ^2^)] = 0.079
*wR*(*F* ^2^) = 0.209
*S* = 1.16
1717 reflections
150 parameters
2 restraints
H atoms treated by a mixture of independent and constrained refinement
Δρ_max_ = 2.49 e Å^−3^
Δρ_min_ = −1.05 e Å^−3^

### 

Data collection: *APEX2* (Bruker, 2012 ▶); cell refinement: *SAINT* (Bruker, 2012 ▶); data reduction: *SAINT*; program(s) used to solve structure: *SHELXS97* (Sheldrick, 2008 ▶); program(s) used to refine structure: *SHELXL97* (Sheldrick, 2008 ▶); molecular graphics: *ORTEP-3 for Windows* (Farrugia, 2012 ▶); software used to prepare material for publication: *WinGX* (Farrugia, 2012 ▶) and *PLATON* (Spek, 2009 ▶).

## Supplementary Material

Crystal structure: contains datablock(s) I, global. DOI: 10.1107/S160053681400155X/su2690sup1.cif

Structure factors: contains datablock(s) I. DOI: 10.1107/S160053681400155X/su2690Isup2.hkl

CCDC reference:

Additional supporting information:  crystallographic information; 3D view; checkCIF report

## Figures and Tables

**Table 1:** Hydrogen-bond geometry (Å, °) *Cg*1 is the centroid of the C2–C7 ring.

*D*—H⋯*A*	*D*—H	H⋯*A*	*D*⋯*A*	*D*—H⋯*A*
O4—H42⋯O1	0.82 (5)	1.81 (6)	2.579 (6)	155 (8)
O4—H41⋯O3^i^	0.82 (2)	2.65 (5)	3.395 (8)	152 (8)
C9—H9⋯O3^ii^	0.93	2.45	3.311 (8)	154
C7—H7⋯*Cg*1^iii^	0.93	2.62	3.395 (6)	141

Symmetry codes: (i) 
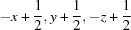
; (ii) 
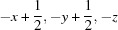
; (iii) 
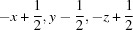
.

**Fig. 1.**
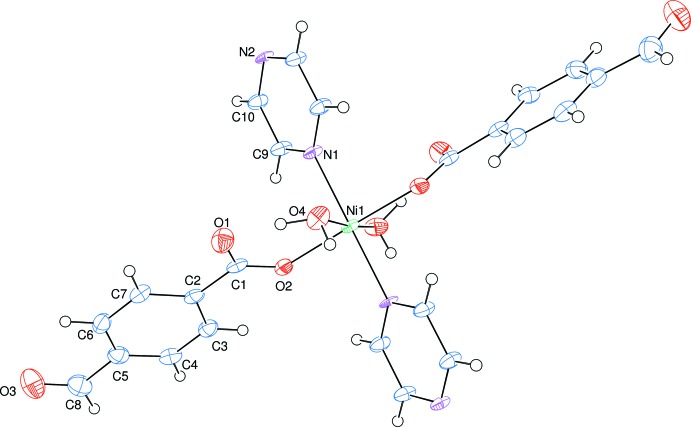
A view of the coordination geometry around the NiII atom of the title molecule, with the atom-labelling. Displacement ellipsoids are drawn at the 50% probability level. The two-fold rotation axis bisects atoms Ni1, N1 and N2.

**Fig. 2.**
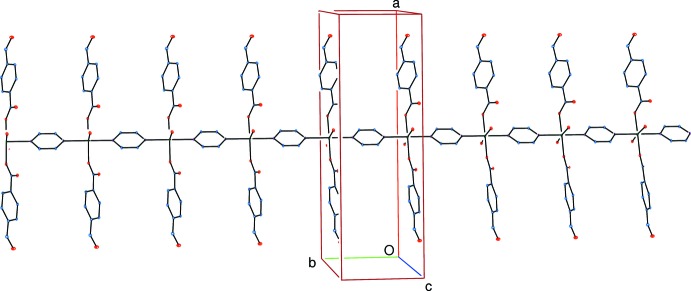
A partial view along the c axis of the crystal packing of the title compound. Hydrogen atoms have been omitted for clarity.

## supplementary crystallographic information

### 1. Comment

The structural functions and coordination relationships of the arylcarboxylate ion in transition metal complexes of benzoic acid derivatives change depending on the nature and position of the substituent groups on the benzene ring, the nature of the additional ligand molecule or solvent, and the medium of the synthesis (Nadzhafov *et al.*, 1981; Shnulin *et al.*, 1981). Transition metal complexes with biochemically active ligands frequently show interesting physical and/or chemical properties, as a result they may find applications in biological systems (Antolini *et al.*, 1982). Some benzoic acid derivatives, such as 4-aminobenzoic acid, have been extensively reported in coordination chemistry, as bifunctional organic ligands, due to the varieties of their coordination modes (Chen & Chen, 2002; Amiraslanov *et al.*, 1979; Hauptmann *et al.*, 2000). The title compound, which is isotructural with the copper(II) complex (Çelik *et al.*, 2014*a*) was synthesized and its crystal structure is reported on herein.

The asymmetric unit of the title compound contains half a Ni^II^ ion, one formylbenzoate (FB) anion, one water molecule and half of a pyrazine molecule. Atoms Ni1, and N1 and N2 of the pyrazine ligand, are located on a two-fold rotation axis (Fig. 1). The pyrazine ligands bridge adjacent Ni^II^ ions forming polymeric chains running along the *b*-axis direction (Fig. 2). The distances between the symmetry related Ni^II^ ions [Ni1···Ni1^i^; symmetry code: (i) *x*, *y* + 1, *z*] is 6.992 (3) Å.

In the equatorial plane of the Ni^II^, coordination sphere is composed of two carboxylate O atoms (O2 and O2^ii^; symmetry code: (ii) - *x*, *y*, - *z + 1/2*) of two symmetry related monodentate formylbenzoate anions and two N atoms (N1 and N2) of the bridging pyrazine ligand, which is bisected by the two-fold rotation axis. The axial positions are occupied by two O atoms (O4 and O4^ii^) of the coordinated water molecules.

The near equality of the C1—O1 [1.250 (7) Å] and C1—O2 [1.260 (6) Å] bonds in the carboxylate group indicate delocalized bonding arrangement, rather than localized single and double bonds. The Ni—N bond lengths are 2.108 (6) and 2.112 (6) Å, while the Ni—O bond lengths are 2.047 (4) Å (for benzoate oxygen) and 2.107 (4) Å (for water oxygen) close to standard values (Allen *et al.*, 1987). The Ni1 atom is displaced out of the mean-plane of the carboxylate group (O1/C1/O2) by 0.0658 (8) Å. The dihedral angle between the planar carboxylate group and the adjacent benzene ring (C2—C7) is 7.0 (6)°, while the benzene and pyrazine rings are oriented at a dihedral angle of 66.2 (3) °. Strong intramolecular O—H···O hydrogen bonds (Table 1) link the water molecules to the carboxylate oxygens.

In the crystal, O-H_water_···O_water_ hydrogen bonds link adjacent chains into layers parallel to the *bc* plane (Table 1). C—H_pyrazine_···O_formyl_ hydrogen bonds (Table 1) link the layers to form a three-dimensional network. There are also weak C—H···π interactions present (Table 1).

### 2. Experimental

The title compound was prepared by the reaction of NiSO_4_.6H_2_O (1.31 g, 5 mmol) in H_2_O (70 ml) and pyrazine (0.40 g, 5 mmol) in H_2_O (30 ml) with sodium 4-formylbenzoate (1.72 g, 10 mmol) in H_2_O (100 ml) at room temperature. The mixture was filtered and set aside to crystallize at ambient temperature for one week, giving blue prismatic crystals.

### 3. Refinement

Atoms H41 and H42 (for H_2_O) were located in a difference Fourier map and were refined with distance restraints: O-H = 0.82 (2) Å. The C-bound H-atoms were positioned geometrically with C—H = 0.93 Å for aromatic H-atoms, and constrained to ride on their parent atoms, with *U*_iso_(H) = 1.2*U*_eq_(C). Both the highest residual electron density and the deepest hole were found 0.88 Å from atom Ni1.

### Crystal data

**Table:**

[Ni(C_8_H_5_O_3_)_2_(C_4_H_4_N_2_)(H_2_O)_2_]	*F*(000) = 976
*M**_r_* = 473.07	*D*_x_ = 1.652 Mg m^−^^3^
Monoclinic, *C*2/*c*	Mo *K*α radiation, λ = 0.71073 Å
Hall symbol: -C 2yc	Cell parameters from 5837 reflections
*a* = 22.1032 (5) Å	θ = 3.1–28.3°
*b* = 6.9925 (2) Å	µ = 1.08 mm^−^^1^
*c* = 12.3366 (3) Å	*T* = 296 K
β = 94.160 (3)°	Prism, blue
*V* = 1901.68 (8) Å^3^	0.48 × 0.23 × 0.14 mm
*Z* = 4	

### Data collection

**Table:**

Bruker SMART BREEZE CCD diffractometer	1717 independent reflections
Radiation source: fine-focus sealed tube	1554 reflections with *I* > 2σ(*I*)
Graphite monochromator	*R*_int_ = 0.070
φ and ω scans	θ_max_ = 25.3°, θ_min_ = 1.9°
Absorption correction: multi-scan (*SADABS*; Bruker, 2012)	*h* = −26→26
*T*_min_ = 0.743, *T*_max_ = 0.860	*k* = −8→8
9913 measured reflections	*l* = −14→12

### Refinement

**Table:**

Refinement on *F*^2^	Primary atom site location: structure-invariant direct methods
Least-squares matrix: full	Secondary atom site location: difference Fourier map
*R*[*F*^2^ > 2σ(*F*^2^)] = 0.079	Hydrogen site location: inferred from neighbouring sites
*wR*(*F*^2^) = 0.209	H atoms treated by a mixture of independent and constrained refinement
*S* = 1.16	*w* = 1/[σ^2^(*F*_o_^2^) + (0.1238*P*)^2^ + 9.9995*P*] where *P* = (*F*_o_^2^ + 2*F*_c_^2^)/3
1717 reflections	(Δ/σ)_max_ < 0.001
150 parameters	Δρ_max_ = 2.49 e Å^−^^3^
2 restraints	Δρ_min_ = −1.05 e Å^−^^3^

### Special details

**Table:**

Geometry. Bond distances, angles etc. have been calculated using the rounded fractional coordinates. All su's are estimated from the variances of the (full) variance-covariance matrix. The cell esds are taken into account in the estimation of distances, angles and torsion angles
Refinement. Refinement of F^2^ against ALL reflections. The weighted R-factor wR and goodness of fit S are based on F^2^, conventional R-factors R are based on F, with F set to zero for negative F^2^. The threshold expression of F^2^ > 2sigma(F^2^) is used only for calculating R-factors(gt) etc. and is not relevant to the choice of reflections for refinement. R-factors based on F^2^ are statistically about twice as large as those based on F, and R- factors based on ALL data will be even larger.

### Fractional atomic coordinates and isotropic or equivalent isotropic displacement parameters (Å^2^)

**Table:**

	*x*	*y*	*z*	*U*_iso_*/*U*_eq_	
Ni1	0.00000	0.44790 (12)	0.25000	0.0222 (3)	
O1	0.13672 (19)	0.3246 (7)	0.3338 (3)	0.0435 (14)	
O2	0.08397 (17)	0.4511 (5)	0.1900 (3)	0.0294 (11)	
O3	0.3999 (2)	0.3724 (9)	0.0460 (4)	0.0614 (19)	
O4	0.0372 (2)	0.4332 (6)	0.4119 (3)	0.0342 (12)	
N1	0.00000	0.1464 (8)	0.25000	0.0242 (17)	
N2	0.00000	0.7500 (8)	0.25000	0.0260 (19)	
C1	0.1321 (2)	0.3907 (7)	0.2394 (5)	0.0277 (16)	
C2	0.1890 (2)	0.3983 (7)	0.1787 (5)	0.0284 (16)	
C3	0.1889 (3)	0.4908 (8)	0.0787 (5)	0.0300 (17)	
C4	0.2420 (3)	0.5042 (9)	0.0271 (5)	0.0320 (17)	
C5	0.2955 (3)	0.4294 (8)	0.0736 (5)	0.0339 (17)	
C6	0.2958 (3)	0.3342 (8)	0.1733 (5)	0.0338 (17)	
C7	0.2424 (2)	0.3196 (8)	0.2243 (5)	0.0290 (17)	
C8	0.3520 (3)	0.4471 (10)	0.0178 (6)	0.045 (2)	
C9	0.0252 (3)	0.0463 (7)	0.1723 (5)	0.0290 (17)	
C10	0.0250 (2)	0.8494 (7)	0.1733 (4)	0.0280 (17)	
H3	0.15320	0.54320	0.04700	0.0360*	
H4	0.24170	0.56450	−0.04010	0.0380*	
H6	0.33140	0.28130	0.20480	0.0410*	
H7	0.24240	0.25590	0.29040	0.0350*	
H8	0.35070	0.52240	−0.04450	0.0540*	
H9	0.04310	0.11080	0.11700	0.0350*	
H10	0.04300	0.78400	0.11840	0.0340*	
H41	0.040 (4)	0.545 (5)	0.430 (7)	0.06 (3)*	
H42	0.0721 (18)	0.396 (13)	0.407 (7)	0.07 (3)*	

### Atomic displacement parameters (Å^2^)

**Table:**

	*U*^11^	*U*^22^	*U*^33^	*U*^12^	*U*^13^	*U*^23^
Ni1	0.0321 (6)	0.0093 (5)	0.0253 (6)	0.0000	0.0022 (4)	0.0000
O1	0.046 (2)	0.049 (3)	0.036 (2)	0.008 (2)	0.0059 (18)	0.012 (2)
O2	0.032 (2)	0.0188 (19)	0.038 (2)	0.0020 (14)	0.0065 (16)	0.0009 (16)
O3	0.045 (3)	0.085 (4)	0.055 (3)	0.001 (3)	0.010 (2)	−0.006 (3)
O4	0.047 (2)	0.031 (2)	0.024 (2)	0.0063 (19)	−0.0012 (17)	−0.0034 (17)
N1	0.036 (3)	0.011 (3)	0.026 (3)	0.0000	0.005 (2)	0.0000
N2	0.034 (3)	0.006 (3)	0.038 (4)	0.0000	0.003 (3)	0.0000
C1	0.039 (3)	0.012 (2)	0.032 (3)	−0.002 (2)	0.002 (2)	0.000 (2)
C2	0.040 (3)	0.013 (2)	0.032 (3)	−0.002 (2)	0.002 (2)	−0.004 (2)
C3	0.036 (3)	0.022 (3)	0.031 (3)	0.000 (2)	−0.004 (2)	−0.002 (2)
C4	0.047 (3)	0.023 (3)	0.026 (3)	−0.006 (2)	0.003 (2)	0.000 (2)
C5	0.040 (3)	0.026 (3)	0.036 (3)	−0.005 (2)	0.004 (2)	−0.007 (2)
C6	0.036 (3)	0.027 (3)	0.038 (3)	0.005 (2)	0.001 (2)	−0.002 (2)
C7	0.039 (3)	0.021 (3)	0.027 (3)	0.005 (2)	0.003 (2)	0.002 (2)
C8	0.050 (4)	0.044 (4)	0.043 (4)	−0.007 (3)	0.008 (3)	−0.002 (3)
C9	0.045 (3)	0.017 (3)	0.026 (3)	−0.002 (2)	0.009 (2)	0.002 (2)
C10	0.041 (3)	0.016 (3)	0.028 (3)	0.000 (2)	0.010 (2)	−0.004 (2)

### Geometric parameters (Å, º)

**Table:**

Ni1—O2	2.048 (4)	C2—C3	1.393 (8)
Ni1—O4	2.107 (4)	C2—C7	1.384 (7)
Ni1—N1	2.108 (6)	C3—C4	1.378 (9)
Ni1—N2	2.112 (6)	C4—C5	1.379 (9)
Ni1—O2^i^	2.048 (4)	C5—C6	1.398 (9)
Ni1—O4^i^	2.107 (4)	C5—C8	1.474 (9)
O1—C1	1.250 (7)	C6—C7	1.381 (8)
O2—C1	1.260 (6)	C9—C10^ii^	1.377 (7)
O3—C8	1.209 (8)	C3—H3	0.9300
O4—H42	0.82 (5)	C4—H4	0.9300
O4—H41	0.81 (4)	C6—H6	0.9300
N1—C9^i^	1.340 (7)	C7—H7	0.9300
N1—C9	1.340 (7)	C8—H8	0.9300
N2—C10	1.327 (6)	C9—H9	0.9300
N2—C10^i^	1.327 (6)	C10—H10	0.9300
C1—C2	1.511 (7)		
			
O2—Ni1—O4	92.38 (16)	C3—C2—C7	119.4 (5)
O2—Ni1—N1	90.63 (10)	C1—C2—C7	120.0 (5)
O2—Ni1—N2	89.37 (10)	C1—C2—C3	120.5 (5)
O2—Ni1—O2^i^	178.75 (15)	C2—C3—C4	119.6 (6)
O2—Ni1—O4^i^	87.68 (16)	C3—C4—C5	121.0 (6)
O4—Ni1—N1	87.20 (12)	C4—C5—C6	119.7 (6)
O4—Ni1—N2	92.80 (12)	C4—C5—C8	120.2 (6)
O2^i^—Ni1—O4	87.68 (16)	C6—C5—C8	120.0 (6)
O4—Ni1—O4^i^	174.41 (17)	C5—C6—C7	119.1 (6)
N1—Ni1—N2	180.00 (1)	C2—C7—C6	121.2 (6)
O2^i^—Ni1—N1	90.63 (10)	O3—C8—C5	125.7 (7)
O4^i^—Ni1—N1	87.20 (12)	N1—C9—C10^ii^	120.9 (5)
O2^i^—Ni1—N2	89.37 (10)	N2—C10—C9^iii^	122.2 (5)
O4^i^—Ni1—N2	92.80 (12)	C2—C3—H3	120.00
O2^i^—Ni1—O4^i^	92.38 (16)	C4—C3—H3	120.00
Ni1—O2—C1	125.3 (4)	C3—C4—H4	119.00
Ni1—O4—H41	103 (6)	C5—C4—H4	120.00
Ni1—O4—H42	105 (6)	C5—C6—H6	120.00
H41—O4—H42	106 (9)	C7—C6—H6	120.00
C9—N1—C9^i^	117.0 (5)	C2—C7—H7	120.00
Ni1—N1—C9	121.5 (3)	C6—C7—H7	119.00
Ni1—N1—C9^i^	121.5 (3)	O3—C8—H8	117.00
Ni1—N2—C10	121.6 (3)	C5—C8—H8	117.00
Ni1—N2—C10^i^	121.6 (3)	N1—C9—H9	120.00
C10—N2—C10^i^	116.8 (5)	C10^ii^—C9—H9	120.00
O1—C1—O2	125.7 (5)	N2—C10—H10	119.00
O2—C1—C2	116.9 (5)	C9^iii^—C10—H10	119.00
O1—C1—C2	117.4 (4)		
			
O4—Ni1—O2—C1	22.0 (4)	Ni1—N1—C9—C10^ii^	179.9 (4)
N1—Ni1—O2—C1	−65.3 (4)	Ni1—N2—C10—C9^iii^	179.9 (4)
N2—Ni1—O2—C1	114.7 (4)	O1—C1—C2—C3	−172.2 (5)
O4^i^—Ni1—O2—C1	−152.5 (4)	O1—C1—C2—C7	5.2 (8)
O2—Ni1—N1—C9	−35.6 (3)	O2—C1—C2—C3	8.0 (7)
O2—Ni1—N1—C9^i^	144.4 (3)	O2—C1—C2—C7	−174.5 (5)
O4—Ni1—N1—C9	−128.0 (3)	C1—C2—C3—C4	176.9 (5)
O4—Ni1—N1—C9^i^	52.0 (3)	C7—C2—C3—C4	−0.6 (8)
O2^i^—Ni1—N1—C9	144.4 (3)	C1—C2—C7—C6	−176.3 (5)
O4^i^—Ni1—N1—C9	52.0 (3)	C3—C2—C7—C6	1.2 (8)
O2—Ni1—N2—C10	35.5 (3)	C2—C3—C4—C5	−0.8 (9)
O2—Ni1—N2—C10^i^	−144.5 (3)	C3—C4—C5—C6	1.6 (9)
O4—Ni1—N2—C10	127.9 (3)	C3—C4—C5—C8	−179.3 (6)
O4—Ni1—N2—C10^i^	−52.1 (3)	C4—C5—C6—C7	−1.0 (9)
O2^i^—Ni1—N2—C10	−144.5 (3)	C8—C5—C6—C7	179.9 (6)
O4^i^—Ni1—N2—C10	−52.1 (3)	C4—C5—C8—O3	−172.9 (7)
Ni1—O2—C1—O1	−2.3 (8)	C6—C5—C8—O3	6.3 (10)
Ni1—O2—C1—C2	177.5 (3)	C5—C6—C7—C2	−0.4 (9)

Symmetry codes: (i) −*x*, *y*, −*z*+1/2; (ii) *x*, *y*−1, *z*; (iii) *x*, *y*+1, *z*.

### Hydrogen-bond geometry (Å, º)

Cg1 is the centroid of the C2–C7 ring.

**Table:**

*D*—H···*A*	*D*—H	H···*A*	*D*···*A*	*D*—H···*A*
O4—H42···O1	0.82 (5)	1.81 (6)	2.579 (6)	155 (8)
O4—H41···O3^iv^	0.82 (2)	2.65 (5)	3.395 (8)	152 (8)
C9—H9···O3^v^	0.93	2.45	3.311 (8)	154
C7—H7···*Cg*1^vi^	0.93	2.62	3.395 (6)	141

Symmetry codes: (iv) −*x*+1/2, *y*+1/2, −*z*+1/2; (v) −*x*+1/2, −*y*+1/2, −*z*; (vi) −*x*+1/2, *y*−1/2, −*z*+1/2.
